# Significance of Interleukin-6 in Papillary Thyroid Carcinoma

**DOI:** 10.1155/2016/6178921

**Published:** 2016-03-13

**Authors:** Toral P. Kobawala, Trupti I. Trivedi, Kinjal K. Gajjar, Darshita H. Patel, Girish H. Patel, Nandita R. Ghosh

**Affiliations:** Division of Molecular Endocrinology, Cancer Biology Department, The Gujarat Cancer & Research Institute, NCH Compound, Asarwa, Ahmedabad, Gujarat 380016, India

## Abstract

This study sought to reveal the significance of IL-6 in papillary thyroid carcinoma by determining its circulating levels, tumoral protein, and mRNA expressions. As compared to the healthy individuals, serum IL-6 was significantly higher in patients with benign thyroid diseases and PTC. Further, its level was significantly higher in PTC patients as compared to patients with benign thyroid diseases. ROC curves also confirmed a good discriminatory efficacy of serum IL-6 between healthy individuals and patients with benign thyroid diseases and PTC. The circulating IL-6 was significantly associated with poor overall survival in PTC patients. IL-6 immunoreactivity was significantly high in PTC patients as compared to the benign thyroid disease patients. Significantly higher IL-6 mRNA expression was also observed in the primary tumour tissues of PTC patients than the adjacent normal tissues. The protein expression of IL-6 at both the circulating and tissue level correlated with disease aggressiveness in PTC patients. Moreover, a significant positive correlation was observed between the IL-6 protein and mRNA expression in the primary tumours of PTC patients. Finally in conclusion, IL-6 has an important role in thyroid cancer progression. Thus targeting IL-6 signalling can help in clinical management of thyroid carcinoma patients.

## 1. Introduction

IL-6 is apleiotropic cytokine having a central role in the regulation of inflammatory and immune responses [1]. It is secreted by different cell types including macrophages, T and B lymphocytes, fibroblasts, endothelial cells, and cancer cells [2]. It was cloned in 1986 as the B-cell differentiation factor [3].

IL-6 has been known to exert its biological activities through binding to its receptors and further leads to activation of signal transduction via various pathways like: janus kinase/signal transducers and activators of transcription (JAK/STAT), phosphatidylinositol 3′ kinase/Akt (PI3K/Akt), and mitogen activated protein kinase (MAPK) pathway [4]. Numerous studies indicate that IL-6 and its related signalling pathways have been identified to contribute to proliferation, migration, and invasion of various tumour cells [5–9] and its expression is associated with poor prognosis in many types of cancers [10–12]. Moreover, the physiological role of IL-6 has been shown to promote not only tumour proliferation, but also metastasis and symptoms of cachexia [6, 13, 14]. Increased expression of IL-6 has been reported in different types of cancers and high serum levels of IL-6 have been associated with metastasis and unfavourable prognosis [2, 15–17]. Further, possible involvement of IL-6 signalling in the resistance to chemotherapy and radiotherapy has been documented in few studies [18–20]. Regarding its therapeutic role, blockade of IL-6 in the various autoimmune and inflammatory diseases, by tocilizumab, a humanized anti-(human IL-6R) monoclonal antibody, has proved to improve symptoms of rheumatoid arthritis, Castleman's disease, and systemic juvenile idiopathic arthritis [21]. IL-6 signalling has also been investigated as a potential target for several types of cancer therapies [22, 23].

Thyroid cancer is the most common and slowly progressing endocrine malignancy, accounting for less than 1% of malignancies diagnosed. Although survival is generally good, the mortality rate is higher than all other endocrine organ cancers. It was estimated that 6 out of every 1 million people die due to thyroid cancer. About 85% of thyroid cancers are papillary thyroid cancer (PTC). Despite high survival rates, local recurrence and metastases may occur in some patients and this may require a more aggressive surgical treatment [24]. Moreover, an association between the thyroid cancer and a history of underlying inflammatory conditions of benign diseases has been evident from the literature. Besides this, very often, a pathologist is confronted with thyroid lesions in which the distinction between benign and malignant can be rather difficult and as a result, the decision supporting one or another has clinical consequences and implies different treatment modalities. Thus, it was hypothesised that IL-6, a proinflammatory cytokine, and one of the chief components of the underlying inflammatory conditions, may help in differential diagnosis of thyroid diseases.

Thus, the goal of this study was to examine the role of IL-6 in benign and papillary thyroid cancer patients and correlate the results with clinicopathological parameters and disease outcome of PTC patients. To achieve this aim, we assessed the circulating levels of IL-6 in both patients with benign thyroid diseases and PTC and compared with healthy individuals. We further studied the protein expression of IL-6 in the primary tumours of PTC patients in relation to the benign tissues and also determined its mRNA expression in the primary tumour as well as adjacent normal tissues of histologically confirmed PTC patients.

## 2. Materials and Methods

### 2.1. Patients

Sixty-seven patients with benign thyroid diseases and 83 pathologically confirmed PTC patients were included in this study. This patient group and their clinicopathological features shown in Table 1 are same as mentioned previously [25]. Forty-five out of 67 benign thyroid disease patients underwent surgery at our institute and were included for immunohistochemical analysis. The WHO classification and the AJCC/UICC TNM staging system were used to histopathologically classify the tumours and to stage the thyroid cancer patients, respectively. Accordingly, the patients were grouped into younger (<45 years) and elder (≥45 years) age groups. All patients were followed for a period of 4 years or until death within that period. Complete follow-up details were obtained in 92% (76/83) PTC patients and were included for overall survival (OS) analysis. Nine percent (7/76) patients amongst these had persistent disease and hence were not included for the disease free survival (DFS) analysis. Therefore, 69/76 PTC patients were included for DFS analysis. IL-6 mRNA was determined in the primary tumour and adjacent normal tissues of sixty PTC patients. With respect to IL-6 mRNA expression, DFS was evaluated in 54/60 PTC patients as the rest of six patients had persistent disease and hence were not included for the DFS analysis, while all sixty patients were included for OS analysis.

### 2.2. Sample Collection

This study has been approved by Institutional Scientific and Ethical Committees and informed consent was obtained from all patients prior to sample collection. To detect the circulating levels of IL-6, pretherapeutic blood samples were collected from all patients as well as from 67 healthy individuals. Serum was separated after centrifugation and was preserved at −80°C until analysis. Primary tissue samples of patients were collected on ice directly from the operation theatre. Both tumour and adjacent normal tissue were selected by a pathologist and divided into two portions. One portion was submitted for routine histopathological evaluation and the other portion was snap frozen in liquid nitrogen and preserved at −80°C for total RNA extraction. For Immunohistochemistry, paraffin embedded tissue blocks of all the patients (who underwent surgery) were retrieved from the Histopathology Department of our institute. The clinical and histopathological details of the patients were noted from the case files maintained at the Medical Record Department of the institute.

### 2.3. Circulating Levels of IL-6 by Enzyme Linked Immunosorbent Assay (ELISA)

The circulating levels of IL-6 were estimated from the serum samples using commercially available ELISA kit from Krishgen Biosystems following manufacturer's instructions. The unknown concentrations were interpreted from the standard curve generated in Graphpad prism 5 software.

### 2.4. Tumoral Protein Expression of IL-6 by Immunohistochemistry (IHC)

Immunohistochemical staining was performed for detection of tumoral expression of IL-6 in primary tumours of PTC patients and in patients with benign thyroid diseases. Briefly, 3–5 *μ*m thick sections were cut from the formalin fixed paraffin embedded tissue blocks using Leica microtome and mounted on APES coated glass slides. The immunohistochemical staining was carried out using primary mouse monoclonal IL-6 antibody from R&D Systems (MAB2061) and MACH4 Universal HRP-Polymer Detection System from Biocare Medicals, USA, as per manufacturer's protocol recommendations. Antigenicity was retrieved by heating the sections in 10 mM sodium citrate buffer (pH, 6.0) for 20 mins in a pressure cooker prior to application of the primary antibody. All the sections were scored independently by two individual observers in a blinded fashion. A semiquantitative Immunoreactive Score (IRS) method of Remmele and Stegner [26] based on staining positivity and staining intensity was implemented. Staining positivity was scored as 0 for no stained cells, 1 for staining in 1% to 10% of cells, 2 for staining in 11% to 50% of cells, 3 for staining in 50% to 80% of cells, and 4 for staining in >80% of cells. The staining intensity was scored as 0 for no staining, 1 for weak/faint staining, 2 for moderate staining, and 3 for intense/dark staining. The IRS score was then obtained by multiplying the staining positivity and the staining intensity and therefore, theoretically the scores could range from 0 to 12. For statistical evaluation, the median IRS in the two subgroups of patients was used as cut-off value to divide the patients into low (≤median IRS) and high (>median IRS) expression groups, respectively.

### 2.5. IL-6 mRNA Expression by Reverse Transcriptase- Polymerase Chain Reaction (RT-PCR)

The total RNA was extracted by guanidine thiocyanate-phenol-chloroform extraction method modified from that by Chomczynski and Sacchi [27] and quantitated spectrophotometrically at 260 nm and 280 nm (Helios *α*, Thermo Spectronic, UK). The integrity of the RNA was confirmed by on 1.4% agarose gel. RT-PCR was performed using the OneStep RT-PCR kit (Qiagen, USA) to amplify IL-6 mRNA. Primers 5′-ATG TAG CCG CCC CAC ACA GA-3′ (sense) and 5′-GCA TCC ATC TTT TTC AGC CAT C-3′ (antisense) were used to amplify a 191 bp fragment specific for IL-6. The housekeeping GAPDH mRNA was used as an internal control. Primers 5′-CGG AGT CAA CGG ATT TGG TCG TAT-3′ (sense) and 5′-AGC CTT CTC CAT GGT GGT GAA GAC-3′ (antisense) were used to amplify a 306 bp fragment specific for GAPDH. 1 *μ*g of total RNA was added per reaction. RT-PCR was performed in Mastercycler gradient (Eppendorf, Germany) using the following conditions: IL-6: 60°C for 30 minutes, 95°C for 15 minutes, followed by 36 cycles of 94°C for 45 seconds, 58.1°C for 45 seconds, and 72°C for 1 minute and final extension at 72°C for 10 minutes; and GAPDH: 60°C for 30 minutes, 95°C for 15 minutes, followed by 36 cycles of 94°C for 45 seconds, 60°C for 45 seconds, and 72°C for 1 minute and final extension at 72°C for 10 minutes. The amplified products were run on 2% agarose gels and the intensity of the products was measured as counts/mm^2^ and integrated on gel documentation system (Alpha Innotech, USA) using densitometric analysis.

### 2.6. Statistical Analysis

The data were analyzed statistically using the Statistical Package for Social Sciences (SPSS) software version 16 (SPSS Inc., USA). Independent samples *t*-test was used to compare the means of circulating levels between two groups of subjects and also to assess the association of the analytes with the clinicopathological parameters of thyroid cancer patients. Receiver's operating characteristic (ROC) curve was constructed to determine the discriminating efficacy of the circulating IL-6 between healthy individuals and patients with thyroid diseases. Two-tailed *χ* ^2^ test was used to compare the tumoral protein expressions in benign and carcinoma patients and also to determine association between protein expression and clinicopathological parameters of carcinoma patients. In case of less than five patients in the cells of 2 × 2 tables, Yate's continuity correction value along with its two-tailed significance was taken into consideration. Wilcoxon signed ranks test for two-related samples was executed to compare the mRNA expressions from the malignant and corresponding adjacent normal tissues of carcinoma patients, while the correlation of mRNA expression with clinicopathological parameters was analyzed by Mann Whitney *U*-test. Correlation between two parameters was calculated using Spearman's correlation coefficient (*r*) method. Univariate survival analysis was evaluated using Kaplan-Meier method and log rank test was used to analyze difference in survival curves and to assess the prognostic significance of DFS and OS. Multivariate survival analysis was completed using Cox forward step-wise regression model. *P* values ≤ 0.05 were considered to be significant.

## 3. Results

### 3.1. Circulating Levels, Tumoral Protein, and mRNA Expression of IL-6

The circulating levels were expressed as Mean ± Standard Error (M ± SE). As compared to the healthy individuals, serum IL-6 was significantly higher in both patients with benign thyroid diseases (*P* = 0.004) and PTC (*P* = 0.002). Further, its level was also significantly higher in PTC patients as compared to the patients with benign thyroid diseases (*P* = 0.007) (Table 2).

**Figure 1:**
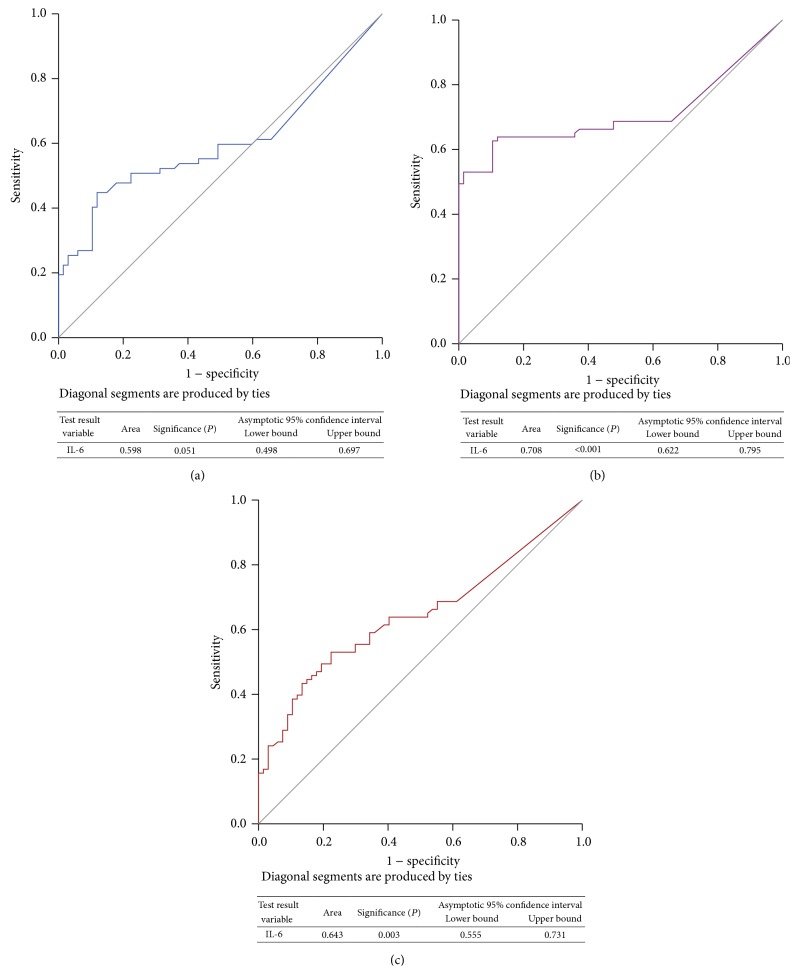
(a) ROC curve for IL-6 in healthy individuals versus patients with benign thyroid diseases. (b) ROC curve for IL-6 in healthy individuals versus patients with papillary thyroid carcinoma. (c) ROC curve for IL-6 in patients with benign thyroid diseases versus papillary thyroid carcinoma.

The ROC curves also confirmed that serum IL-6 exhibited a good discriminatory efficacy between healthy individuals and patients with benign thyroid diseases (AUC = 0.598, *P* = 0.051) (Figure 1(a)) and also between healthy individuals and PTC patients (AUC = 0.708, *P* < 0.001) (Figure 1(b)). Additionally, the circulating IL-6 levels could also significantly differentiate PTC patients from patients with benign thyroid diseases (AUC = 0.643, *P* = 0.003) (Figure 1(c)).

**Figure 2:**
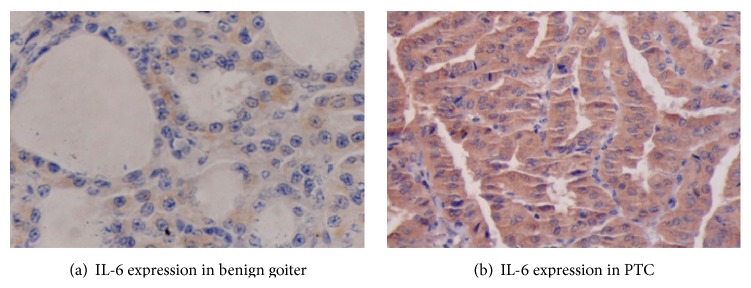
Representative staining patterns of IL-6 expression in primary tumours of patients with benign thyroid diseases and PTC.

**Figure 3:**
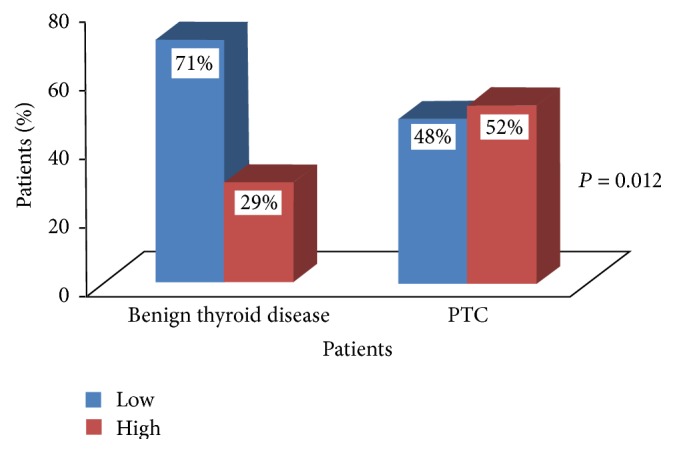
Expression of IL-6 in benign thyroid disease and PTC patients.

The expression of IL-6 protein was localized in cytoplasm of the thyroid follicular cells. Its expression was detectable in 87% (39/45) of the patients with benign thyroid diseases and IRS-4 was the median score, while in PTC patients it was detectable in 96% (80/83) of tumours with median IRS value as 8. Representative staining of IL-6 protein expression in benign thyroid and PTC lesions is shown in Figures 2(a) and 2(b), respectively. The incidence of immunoreactivity of IL-6 was significantly high in PTC patients as compared to the benign thyroid disease patients. It was noted that IL-6 expression was higher in 52% (43/83) of PTC patients, in comparison to only 29% (13/45) of benign thyroid disease patients exhibiting higher expressions (IL-6: *χ* ^2^ = 6.228, *r* = +0.221, *P* = 0.012) (Table 2, Figure 3).

**Figure 4:**
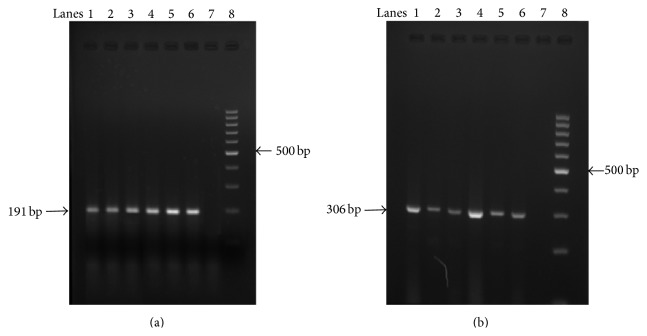
(a) Representative IL-6 mRNA expression. Lanes 1–6: presence of IL-6 mRNA in primary tumours. Lane 7: negative control. Lane 8: 100 bp ladder. (b) Representative GAPDH mRNA expression. Lanes 1–6: presence of GAPDH in primary tumours. Lane 7: negative control. Lane 8: 100 bp ladder.

In PTC patients (*N* = 60), M ± SE of IL-6 mRNA expression in tumour and their adjacent normal tissues were 4969.46 ± 903.77 counts/mm^2^ and 1076.06 ± 301.70 counts/mm^2^, respectively. Using nonparametric Wilcoxon signed ranks test for two-related samples, statistical significant difference was noted in IL-6 mRNA expression between tumour and corresponding adjacent normal tissues with the primary tumour tissues showing considerably higher expression of IL-6 mRNA than the adjacent normal tissues (*P* < 0.001) (Table 2). IL-6 mRNA expression in PTC patients is depicted in Figure 4(a). GAPDH was used as housekeeping gene and Figure 4(b) is the representative picture of GAPDH mRNA expression in PTC patients.

### 3.2. Correlation of IL-6 with Clinicopathological Parameters in PTC Patients

As shown in Table 3, the circulating IL-6 levels were found to be significantly higher in male patients (*P* = 0.016) and in PTC patients having larger tumour size (*P* = 0.043), presence of metastasis (*P* < 0.001), and extrathyroidal extension of tumours (*P* = 0.019) when compared to their respective counterparts. Moreover, the protein expression of IL-6 was found to be significantly higher in patients with larger tumour size (*χ* ^2^ = 4.270; *r* = +0.227; *P* = 0.039), in PTC patients showing capsular invasion of the tumours (*χ* ^2^ = 4.360; *r* = +0.229; *P* = 0.037), and in those having extrathyroidal extension of tumours (*χ* ^2^ = 5.032; *r* = +0.236; *P* = 0.025) in relation to their respective counterparts. Further, the Mann Whitney *U*-test revealed that IL-6 mRNA expression in primary tumours did not show any significant association with any of studied clinicopathological features. On the other hand, the IL-6 mRNA expression in corresponding adjacent normal tissues was significantly higher in cases with smaller tumour size (*P* = 0.037) and in patients with presence of residual disease (*P* = 0.035) as compared to those with larger tumour size and patients showing absence of residual disease, respectively.

### 3.3. Association of IL-6 with Disease Free and Overall Survival in PTC Patients

**Figure 5:**
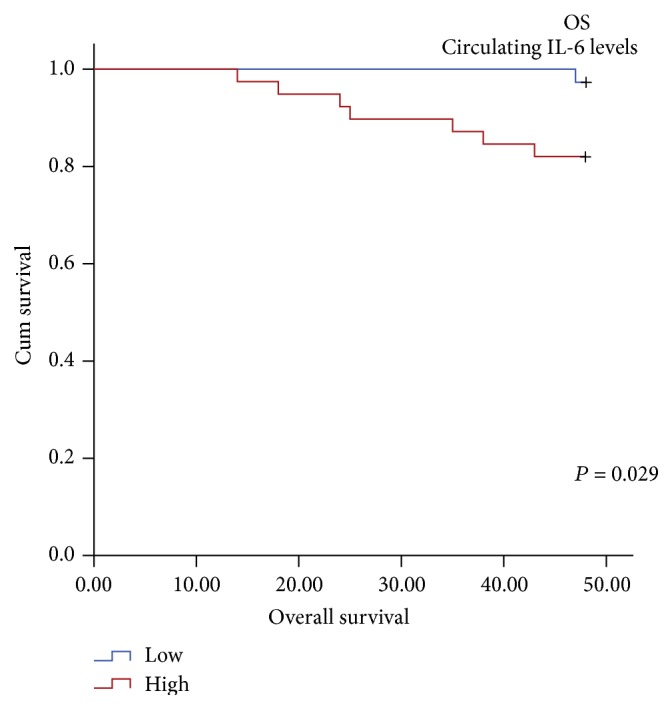
Significantly reduced OS observed in PTC patients with high levels of serum IL-6 as compared to its counterpart.

Kaplan-Meier survival analysis was evaluated for DFS and OS in PTC patients. The median values of IL-6 circulating level, median IRS score for tumoral protein expression, and the median value of IL-6 mRNA expression were used as cut-off value to divide the PTC patients into low (≤median) and high (>median) expression groups, respectively. The difference in survival curves was analyzed using the log rank test. Circulating IL-6 was the significant prognosticator for OS (log rank = 4.77; df = 1; *P* = 0.029) (Table 4(b), Figure 5) while it was not able to predict DFS in the PTC patients. Further, the Kaplan-Meier survival analysis and the log rank test revealed that neither the tumoral protein expression nor the IL-6 mRNA expressions in primary tumours or the adjacent normal tissues were able to predict DFS or OS in the PTC patients (Tables 4(a) and 4(b)).

### 3.4. Intercorrelation between Circulating Levels of IL-6, Its Protein Expression, and mRNA Expression in PTC Patients

In PTC patients, the circulating levels of IL-6 were not significantly associated with its tumoral protein expression (*r* = 0.119, *P* = 0.284) or with the mRNA expression in primary tumour (*r* = −0.130, *P* = 0.322) or adjacent normal tissues (*r* = −0.154, *P* = 0.241). Further, nonparametric Spearman's correlation revealed a significant positive correlation of IL-6 protein expression with the IL-6 mRNA expressions in the primary tumours of PTC patients (*r* = 0.333, *P* = 0.009), while no such association was observed with the IL-6 mRNA expression in adjacent normal tissues (*r* = −0.028, *P* = 0.833) (Table 5).

## 4. Discussion

IL-6 is one of the most widely investigated cytokines in various diseases including cancer. As concerned with its role in thyroid, the present study demonstrated significantly higher levels of circulating IL-6 in benign as well as PTC patients as compared to the healthy individuals. This finding was further supported by the ROC curves which revealed that IL-6 could be one of the potential markers for differentiating patients with benign thyroid diseases, PTC patients from the healthy individuals. Additionally, it could also efficiently discriminate between the patients with benign thyroid diseases and PTC. Similar to present findings, Bartalena et al. reported increased serum IL-6 in multinodular goiter and they considered it as a marker of thyroid destructive inflammatory processes [28]. Recently, Provatopoulou et al. have also reported significant higher levels of serum IL-6 in patients with benign thyroid conditions and thyroid cancer, compared to healthy controls [29]. Also serum IL-6 levels were also found to be elevated in patients with chronic liver disease including cirrhosis and HCC [30]. Giannitrapani et al. have reported that IL-6 levels were significantly higher in HCC patients than that in patients with liver cirrhosis [31]. Similar to this and the current results, Uchiyama et al. have also shown increased levels of serum IL-6 in colorectal cancer as compared to patients with adenoma [32] while Szkaradkiewicz et al. demonstrated that IL-6 levels were elevated in both patients with inflammatory bowel disease and colorectal cancer [33]. Multiple studies have consistently reported that, as compared to the healthy controls, the circulating levels of IL-6 were significantly higher in patients with different malignancies like colorectal cancer [33–36], breast cancer [37–40], pancreatic cancer [41], gastroesophageal cancer [42], gastric cancer [43], and non-small-cell lung carcinoma [44]. Moreover, Sun et al. have also observed that IL-6 was able to promote the proliferation of nasopharyngeal carcinoma [45].

Further, in present study, when serum IL-6 was correlated with various clinicopathological parameters of PTC patients, it was noted that levels of IL-6 showed a significant positive correlation with larger tumour size, presence of distant metastasis at the time of diagnosis, and extrathyroidal extension of tumours. Moreover, male patients had predominantly higher levels of IL-6 than the female patients. In their studies, Salgado et al., Goswami et al., Dethlefsen et al., Tripsianis et al., and Sanguinetti et al. have suggested that IL-6 assumes a role in the upregulation of malignant characteristics in breast cancer cells and that high IL-6 serum levels are associated with poor outcome in breast cancer patients [40, 46–49]. Data from epidemiological studies is also accruing in support of a contributory role of elevated circulating IL-6 in patients with advanced tumour stages and aggressive behaviour of various cancers, such as non-small-cell lung cancer, colorectal cancer, breast cancer, and renal cell carcinoma [11, 35, 39, 50–54]. IL-6 has been implicated as an autocrine promoter of cancer growth for various human cancers such as biliary tract epithelial cancers, multiple myeloma, and prostate cancer [55, 56]. Knüpfer and Preiss revealed that higher IL-6 levels were significantly associated with tumour size, metastasis, stage, and decreased survival in colorectal cancer patients [34] and Ahmed et al. have demonstrated its association with tumour size in breast cancer patients [37]. It can be suggested through these observations that circulating IL-6 may be partly derived from spillover of tumour produced IL-6 which hereby explains the association of high IL-6 levels with larger tumour size and presence of extrathyroidal extension of tumours.

The present study also demonstrates that higher circulating level of IL-6 was significantly associated with poor OS in PTC patients. Nakashima et al. have reported IL-6 to be independently associated with survival in prostate cancer patients [57]. Its role as an independent negative prognostic factor in patients with lymphoma has been confirmed by El Far et al. [58], and Lai et al. reported that IL-6 may be a prognostic marker in patients with Hodgkin disease or B-chronic lymphocytic leukemia [59]. Schultz et al. have demonstrated circulating IL-6 as an independent prognostic biomarker of OS in patients with locally advanced or metastatic pancreatic cancer [60]. IL-6 plays a key role as prognostic factor even in gastric cancer invasion and metastasis and its elevated level in circulation predicts shorter survival [61, 62]. Researchers have observed that serum IL-6 concentration was associated with the progression, histological grade, and bowel wall invasion [63, 64] as well as tumour size and shorter survival periods of colorectal cancer [65]. Recently, Lu et al. have suggested the use of IL-6 concentration in the serum as an indicator of the possibility of colorectal cancer recurrence [66].

Further, the current study observed significant higher cytoplasmic expression of IL-6 protein in the primary tumours of PTC patients than in the patients with benign thyroid disease patients. The overexpression of IL-6 in PTC patients was significantly and linearly correlated with larger tumour size, presence of capsular invasion, and extrathyroidal extension of tumours. Consistent with the present finding, cytoplasmic staining pattern for IL-6 expression was also observed in ovarian cancer [67], renal cell carcinoma [68], colorectal cancer [50], and gastric cancer [61]. Expressions of IL-6 have also been observed to be higher in the primary tumour tissues than the adjacent normal tissues in prostate cancer [69], breast cancer [70], and esophageal squamous cell carcinoma [71, 72] as well as gastric cancer [62, 73]. Moreover, Depner et al. observed that IL-6 expression was strongly upregulated upon progression from benign tumours to highly malignant, metastasizing human skin squamous cell carcinoma [74]. Increased expression of IL-6 immunoreactivity in oral squamous cell carcinoma was more frequently observed in patients with advanced stage, cervical lymph node metastasis, or distant metastasis [75]. Moreover, Il-6 expression was significantly increased in advanced stage as compared to early stage of gastric cancer [73] and colorectal carcinoma [50]. However, Paule et al. found no significant difference in tumour size or grade between renal cell carcinomas with or without expression of IL-6 [68]. Moreover in the present study, IL-6 protein expression did not have a definite role in prognosis of the PTC patients. Similar to current observation, IL-6 expression in the primary tumours of patients with ovarian cancer and gall bladder cancer failed to show significant correlations with prognosis [67, 76]. Contradictory to this, IL-6 expression has been found to be significantly associated with poor prognosis in OSCC patients [75, 77], esophageal squamous cell carcinoma [72], colorectal cancer [50], and ovarian cancer [23]. In a study on colorectal cancer by Chung et al., the tissue expression of IL-6 did not correlate with the serum IL-6 levels, which is comparable to the present finding [50].

In addition to this, in the present study, the IL-6 mRNA expression was higher in the primary tumour tissues of PTC patients as compared to the corresponding adjacent normal tissues. Similar results have been reported in esophageal squamous cell carcinoma [71] and colorectal cancer [32, 66]. Moreover, Sanguinetti et al. have shown that mammospheres from node invasive breast cancer tissues express IL-6 mRNA at higher levels than the mammospheres from matched nonneoplastic mammary glands [49]. In the present study, the mRNA levels in the primary tumours were not associated with any of the clinicopathological parameters or with prognosis in PTC patients but its levels in the corresponding adjacent normal tissues were significantly higher in the PTC patients having smaller tumour size and presence of residual disease after surgery. This may be because although apparently looking normal, biologically there might be some malignant changes in the adjacent normal tissues of such patients and the concept of field cancerization may follow here. Moreover, a significant positive correlation was observed between the IL-6 protein and mRNA expression in the primary tumours of PTC patients. Data reported by Basolo et al. and Ruggeri et al. support that IL-6 tissue expression is related to aggressiveness in PTC [78, 79]. Further, Basolo et al. had shown that downregulation of IL-6 expression may represent a marker of undifferentiated thyroid carcinoma [78]. Moreover, it has also been revealed that certain polymorphisms in the genes encoding IL-6 have been associated with the risk of PTC [80]. Currently, humanized anti- IL-6 receptor monoclonal antibody tocilizumab has been adopted as a first-line biologic therapy for treatment of moderate-to-severe rheumatoid arthritis and for Castleman's disease.

## 5. Conclusion

From the present study, it can be evident that IL-6 has a significant role in thyroid cancer progression and targeting IL-6 signalling can be helpful in clinical management of thyroid carcinoma patients with more aggressive tumour characteristics.

## Figures and Tables

**Table 1:** Clinicopathological characteristics of PTC patients.

Characteristics	*N* (%)	Characteristics	*N* (%)
Age		Bilaterality	
<45 years	41 (49)	Unilateral	61 (74)
≥45 years	42 (51)	Bilateral	22 (26)
Gender		Hemorrhagic area	
Female	56 (68)	Absent	72 (87)
Male	27 (32)	Present	11 (13)
Tumour size		Necrosis	
T1 (*N* = 16) + T2 (*N* = 22)	38 (46)	Absent	67 (81)
T3 (*N* = 30) + T4 (*N* = 15)	45 (54)	Present	16 (19)
Nodal status		Calcification	
Absent	30 (36)	Absent	32 (39)
Present	53 (64)	Present	51 (61)
Metastasis		Extrathyroidal extension	
Absent	73 (88)	Absent	52 (63)
Present	10 (12)	Present	31 (37)
Stage		Fibrosis	
Early [stage I (*N* = 37) + stage II (*N* = 12)]	49 (59)	Absent	61 (74)
Advanced [stage III (*N* = 11) + stage IV (*N* = 23)]	34 (41)	Present	22 (26)
Lymphatic permeation		Inflammation	
Absent	67 (81)	Absent	46 (55)
Present	16 (19)	Present	37 (45)
Vascular permeation		Differentiation	
Absent	74 (89)	Well	76 (92)
Present	09 (11)	Moderate/poor	07 (08)
Capsular invasion		Multifocality	
Absent	55 (66)	Absent	64 (77)
Present	28 (34)	Present	19 (23)
Encapsulation		Residual disease	
Well encapsulated	76 (92)	Absent	24 (29)
Partially/not encapsulated	07 (08)	Present	59 (71)

Treatment			
Surgery	29 (35)		
Surgery + RIA and/RT	54 (65)	Surgery + RIA	50 (60)
		Surgery + RIA + RT	04 (05)

Disease status			
Recurrence/distant metastasis (*N* = 69)		Alive/dead (*N* = 76)	
Absent	62 (90)	Alive	68 (89)
Present	07 (10)	Dead	08 (11)
Recurrence	3 (4)		
Distant metastasis	4 (6)		
Bone	1 (1.5)		
Lung	2 (3.0)		
Bone + lung	1 (1.5)		

**Table 2:** Circulating levels, tumoral protein, and mRNA expression of IL-6.

IL-6	Healthy individuals	Benign thyroid diseases		Papillary thyroid carcinoma (PTC)	
Circulating levels M ± SE (pg/mL)	4.88 ± 0.99	33.73 ± 9.93 *P* = 0.004^*∗*^		246.41 ± 69.41 *P* = 0.002^†^ *P* = 0.007^‡^	

Tumoral protein expression		*Low*	*High*	*Low*	*High*
*N* (%)		32 (71)	13 (29)	40 (48)	43 (52)
		*χ* ^2^ = 6.228; *r* = +0.221; *P* = 0.012^@^			

mRNA expression				*Primary tumour tissues*	*Adjacent normal tissues*
M ± SE (counts/mm^2^)				4969.46 ± 903.77	1076.06 ± 301.70

				*P* < 0.001^∧^	

^**∗**^Significance of circulating IL-6 levels between benign thyroid diseases and healthy individuals.

^†^Significance of circulating IL-6 levels between PTC and healthy individuals.

^‡^Significance of circulating IL-6 levels between PTC and benign thyroid diseases.

^@^Significance of tumoral protein expression of IL-6 between PTC and benign thyroid diseases.

^∧^Significance of IL-6 mRNA expression between primary tumour tissues and adjacent normal tissues in PTC patients.

**Table 3:** Correlation of IL-6 with clinicopathological parameters in PTC patients.

Parameter	Circulating levels		Tumoral protein expression			mRNA expression (adjacent normal tissues)		
	Mean ± SE (pg/mL)	*P*	Low *N* (%)	High *N* (%)		Mean ± SE (counts/mm^2^)	Mean rank	*P*
Gender								
Female	131.33 ± 36.57	**0.016**						
Male	485.07 ± 193.91							
Tumour size								
Small (T1 + T2)	94.08 ± 37.52	**0.043**	23 (61)	15 (39)	*r* = +0.227	1611.52 ± 633.60	35.52	**0.037**
Large (T3 + T4)	375.03 ± 121.41		17 (38)	28 (62)	*P* = 0.039	693.60 ± 240.93	26.91	
Metastasis								
Absent	157.17 ± 39.51	**<0.001**						
Present	897.83 ± 468.64							
Capsular invasion								
Absent			31 (56)	24 (44)	*r* = +0.229			
Present			9 (32)	19 (68)	*P* = 0.037			
Extrathyroidal extension								
Absent	121.81 ± 34.33	**0.019**	30 (58)	22 (42)	*r* = +0.236			
Present	455.04 ± 171.98		10 (32)	21 (68)	*P* = 0.025			
Residual disease								
Absent						1101.40 ± 785.08	24.43	**0.035**
Present						1113.40 ± 237.31	33.54	

*r*: correlation coefficient.

**(a):** 

IL-6	*N*	Patients relapsed *N* (%)	Log rank test statistics
Circulating levels	**69**		
Low	37	3 (8)	Log rank = 0.330, df = 1, *P* = 0.565
High	32	4 (12)	
Tumoral protein expression	**69**		
Low	35	2 (6)	Log rank = 1.480, df = 1, *P* = 0.224
High	34	5 (15)	
mRNA expression (primary tumour tissues)	**54**		
Low	25	2 (8)	Log rank = 0.119, df = 1, *P* = 0.730
High	29	3 (10)	
mRNA expression (adjacent normal tissues)	**54**		
Low	32	3 (9)	Log rank = 0.003, df = 1, *P* = 0.958
High	22	2 (9)	

**(b):** 

IL-6	*N*	Patients died *N* (%)	Log rank test statistics
Circulating levels	**76**		
Low	37	1 (3)	Log rank = 4.772, df = 1, *P* = 0.029
High	39	7 (18)	
Tumoral protein expression	**76**		
Low	38	4 (10)	Log rank = 0.001, df = 1, *P* = 0.977
High	38	4 (10)	
mRNA expression (primary tumour tissues)	**60**		
Low	30	5 (17)	Log rank = 1.496, df = 1, *P* = 0.221
High	30	2 (7)	
mRNA expression (adjacent normal tissues)	**60**		
Low	34	3 (9)	Log rank = 0.748, df = 1, *P* = 0.387
High	26	4 (15)	

**Table 5:** Intercorrelation between circulating levels of IL-6, its protein expression, and mRNA expression in PTC patients.

IL-6 mRNA expression	Circulating IL-6 levels	IL-6 protein expression
Primary tumours	*r* = −0.130 *P* = 0.322	*r* = 0.333 *P* = 0.009
Adjacent normal tissues	*r* = −0.154 *P* = 0.241	*r* = −0.028 *P* = 0.833
	*r* = 0.119, *P* = 0.284	

*r*: correlation coefficient.
